# Asthma in athletes: diagnostic challenges and management strategies

**DOI:** 10.1097/ACI.0000000000001165

**Published:** 2026-06-09

**Authors:** Andrea Giovanni Ledda, Michele Ruggiu, Martina Bullita, Giulia Costanzo, Stefano Del Giacco

**Affiliations:** Department of Medical Science and Public Health, University of Cagliari, Cagliari, Italy

**Keywords:** asthma, exercise-induced asthma, exercise-induced bronchoconstriction, inhaled corticosteroids

## Abstract

**Purpose of review:**

Exercise-induced bronchoconstriction (EIB) and exercise-induced asthma (EIA) are common conditions among athletes and represent an important clinical issue with potential implications for both respiratory health and athletic performance. The prevalence of asthma in athletes is higher than in the general population, likely due to the combined effects of increased ventilatory demand and environmental exposures.

**Recent findings:**

The pathophysiology of EIB is multifactorial and involves osmotic and thermal mechanisms related to airway dehydration during exercise-induced hyperventilation. Although some athletes present a classical T2-high inflammatory phenotype associated with allergic sensitization, a considerable proportion develop EIB through non-T2 inflammatory pathways, including airway epithelial injury and autonomic dysregulation.

**Summary:**

Diagnosis requires objective assessment through pulmonary function testing and bronchial provocation tests. Biomarkers like fractional exhaled nitric oxide (FeNO) and blood eosinophil counts can support phenotypic characterization, but they do not replace functional testing. Management in athletes follows established asthma guidelines and includes inhaled corticosteroids as the cornerstone of therapy, combined with pharmacological and non-pharmacological strategies. Considering anti-doping regulations is also essential to ensure appropriate treatment and compliance.

## INTRODUCTION

Asthma is a chronic inflammatory disorder of the airways characterized by variable respiratory symptoms (such as cough, wheeze, chest tightness and dyspnoea) and variable expiratory airflow limitations, objectively confirmed by lung function testing [1]. The prevalence varies among age groups and ethnicities, but in general it affects approximately 339 million people globally, with rates ranging from 4% to 10% in Western countries [2]. Despite significant advances in the pharmacological management of asthma, even when pharmacotherapy is optimized, individuals with asthma still experience a high disease burden that limits physical activity [3]. In athletes, the prevalence varies by sport, level of competition, training environment and genetic factors [4,5] and is consistently higher than in the general population. High-level athletes have an higher prevalence of asthma and related airway disorders compared to non-athletes, with the presence of airway hyperresponsiveness often even in the absence of symptoms [6^▪▪^]. Among elite athletes, especially those participating in endurance, aquatic and winter sports, prevalence rates for asthma or lower airway dysfunction range from approximately 20% to over 50%, depending on the population studied and the diagnostic methods used. The clinical symptoms of asthma are usually associated with a condition of bronchial hyperreactivity. In athletes, however, the presence of exercise-induced bronchial hyperreactivity has often been documented, regardless of the existence of clinical asthma [7]. This condition is referred to as exercise-induced bronchoconstriction (EIB), while ‘exercise-induced asthma’ (EIA) is reserved for bronchoconstriction episodes triggered by physical exercise in individuals with a documented history of asthma [8]. Notably, exercise is a common trigger for asthma, and high-intensity training, exposure to allergens, and the inhalation of irritants in specific environments play key roles in the increased prevalence of asthma among athletes, particularly because these factors can exacerbate underlying conditions such as exercise-induced bronchial hyperreactivity and lead to more severe asthma symptoms.

This review will discuss the main diagnostic and management strategies and challenges in EIB and EIA. 

**Box 1:**
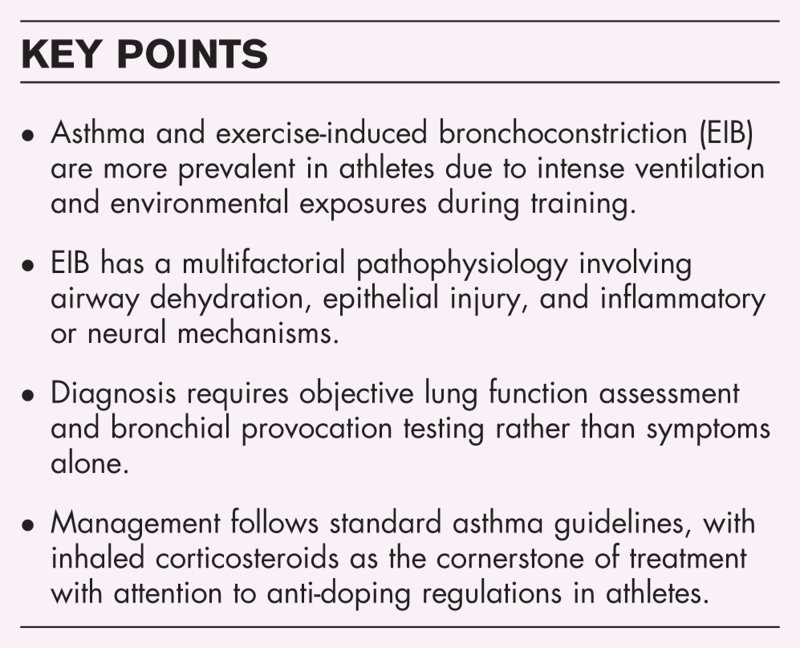
no caption available

## PATHOGENESIS

The increase in airway osmolarity and vasodilation with subsequent airway rewarming are generally considered the main causes of EIB (“osmotic and thermal theories”) [9,10]. Other factors also play a role, such as the type of sport practiced (indoor or outdoor), genetic predisposition, and individual susceptibility. Intense physical activity may induce a transient status of immune downregulation with a shift towards a relatively prevalent T2-high response, clinically associated with an increased prevalence of atopy [11]. Despite the considerable presence of athletes with allergic-phenotype EIB, who develop bronchial obstruction after exercise and exhibit elevated T2 inflammatory markers, EIB can also occur in athletes without evidence of atopic sensitization. In non-T2-high EIB phenotypes, the pathogenic mechanism appears to be mainly related to direct bronchial epithelial damage induced by exercise [12]. A direct injury of the bronchial epithelium might also be caused by viral upper respiratory tract infections [13]. The epithelium damage may lead to the presence of inflammatory mediators in sputum (e.g., interleukin-8) [14] and in serum (e.g., nerve growth factor) [15]. Furthermore, the evidence that CC16 proteins, secreted by club cells in the distal bronchioles to protect the respiratory tract against oxidative stress and inflammation, are increased in urine and serum following an exercise challenge, confirming the role of the small airways in the pathogenesis of EIB [16]. An alternative, non-inflammatory mechanism, measured autonomic dysregulation with an enhanced parasympathetic response (measured hearth rate variability and pupillometry), has been shown to significantly relate to EIB [17].

## DIAGNOSIS

**FIGURE 1:**
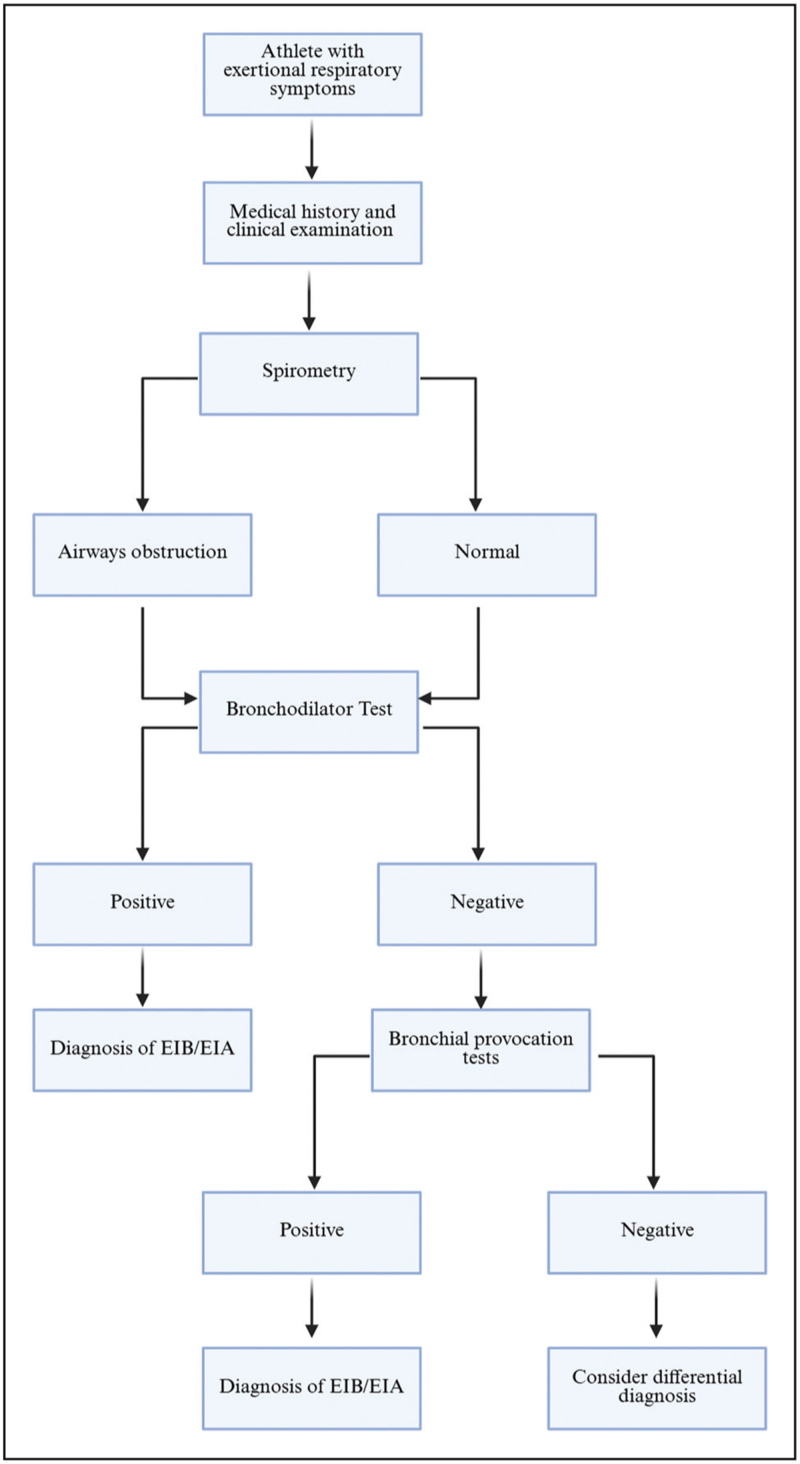
Algorithm of EIB/EIA diagnosis. EIB, exercise-induced bronchoconstriction; EIA, exercise-induced asthma. Created in BioRender. Ledda AG (2026) https://BioRender.com/3kdfr8p.

Objective tests, including spirometry and, if necessary, bronchial provocation tests, should be performed on every athlete who exhibits symptoms that are suggestive of asthma or EIB. It is not possible to perform a diagnosis solely based on the clinical presentation, as research has shown that this method is frequently inaccurate; therefore, it is essential to incorporate objective testing results to confirm the diagnosis of asthma or EIB. Instead, the diagnosis should be based on objective changes in respiratory function that are brought about by physical activity and, in particular, variable airway obstruction via a bronchodilator reversibility test or bronchoprovocation test. The bronchodilator reversibility test is mandatory even when the baseline spirometry is normal, as athletes typically have higher forced expiratory volume in the first second (FEV1) values than the general population [18,19]. A change in FEV1 and forced vital capacity (FVC) after bronchodilator responsiveness (BDR) >10% of the predicted value is considered a positive response [20]. If the bronchodilator test is negative, it is mandatory to perform a provocation test, which can be direct or indirect. Indirect tests, such as exercise challenge, eucapnic voluntary hyperpnea (EVH) and hyperosmolar aerosols (e.g., mannitol or hypertonic saline), induce airway narrowing through inflammatory mediator release and are generally preferred because they more closely reproduce exercise-related mechanisms. A fall in FEV_1_ ≥10% after exercise or EVH supports a diagnosis of EIB, whereas a ≥15% reduction after hyperosmolar agents suggests bronchoconstriction or asthma with or without EIB. Exercise testing is typically performed at high intensity under controlled environmental conditions with serial post-exercise spirometry, while EVH is considered the gold-standard indirect test despite limited availability. Direct bronchial provocation tests, most commonly using methacholine or histamine, assess airway hyperresponsiveness through direct stimulation of muscarinic receptors on airway smooth muscle, leading to bronchoconstriction. Current recommendations favour expressing the results as the provocative dose causing a 20% fall in FEV_1_ (PD20), which improves comparability across devices and protocols compared with concentration-based metrics (PC20). Although the response primarily reflects intrinsic smooth muscle properties, airway structure, baseline calibre, and mechanical tethering forces also influence airflow limitations. Direct tests are therefore useful for identifying underlying asthma or airway hyperresponsiveness but are less sensitive than indirect challenges for diagnosing EIB in athletes, which can lead to missed diagnoses and inadequate management of the pathology. Biomarkers are gaining an emerging role in the diagnosis and phenotyping of asthma. We already stated that in most cases, EIB is sustained by a high T2 pathogenesis, and we introduced the role of CC16. Exhaled fraction of nitric oxide (FeNO) and blood eosinophils are known biomarkers of T2 inflammation that are easy to assess routinely. Studies have shown that values of FeNO ≥40 ppb have a good specificity in EIB diagnosis, while blood eosinophils demonstrated good specificity and positive predictive value. These results highlight the role of T2 inflammation in EIB [22,23] (Fig. 1) [21].

## DIFFERENTIAL DIAGNOSIS

The differential diagnosis of asthma may consider several conditions. The complexity of the differential diagnosis increases with age and mainly concerns master athletes, in whom cardiac diseases, occupational exposures, and other chronic pulmonary conditions are more common. The pathologies can be classified in: Upper Airway obstruction, pulmonary disease, cardiac disease and other conditions (Table 1). Patients with upper airway obstruction can mimic severe asthma, and typically these patients present a localized wheeze and stridor in the large airways [25]. Assessing the flow–volume loop in such patients will reveal a reduction in inspiratory and expiratory flow. Bronchoscopy can demonstrate the site of narrowing in the upper airways. Persistent wheezing auscultation in a localized area of the chest wall may indicate endobronchial obstruction due to lung cancer or a foreign body. Eosinophilic pneumonias and systemic vasculitis, including eosinophilic granulomatosis with polyangiitis and polyarteritis nodosa, may be associated with wheezing, and their systemic clinical manifestations may help in their identification. Chronic obstructive pulmonary disease (COPD) is usually easy to differentiate from asthma. The symptoms in COPD are more persistent, show less variability, are progressive, and usually exhibit minimal reversibility to bronchodilator agents. The literature highlights an “overlap syndrome", where COPD patients have asthma-like features with increased sputum eosinophils and a response to oral corticosteroids [26]. Among the cardiac conditions that can mimic asthma there are left ventricular failure, pulmonary embolism, and severe mitral regurgitation, generally accompanied by orthopnoea, pulmonary oedema, palpitations, arrhythmia and chest pain [27]. Iron deficiency and anaemia reduce haemoglobin and thus oxygen delivery, causing tissue hypoxia, compensatory higher heart rate and ventilation, earlier lactate accumulation, and reduced endurance performance. In athletes, this manifests as breathlessness, fatigue and impaired recovery, especially at higher intensities [28].

**Table 1:** Differential diagnosis of asthma

Upper airways	Pulmonary	Cardiac	Other
Foreign body Upper airway obstruction Vocal cord dysfunction	COPD Pneumonia Pneumothorax Lung cancer Interstitial lung disease EGPA	Angina pectoris Left ventricular failure Mitral valve disease Pulmonary Embolism	Anaemia Iron deficiency GERD Obesity Hyperventilation Physical deconditioning

COPD, chronic obstructive pulmonary disease; EGPA, eosinophilic granulomatosis with polyangiitis; GERD, gastroesophageal reflux disease.

Adapted from Fishman's Pulmonary Diseases and Disorders Fifth Edition, 2015 by McGraw-Hill Education [24].

## TREATMENT AND MANAGEMENT

The treatment of athletes affected by EIB and EIA should follow the same guidelines used for patients with asthma in the general population [8], including the step-up approach recommended by the GINA guidelines in case of worsening symptoms, indicating reduced control of the underlying disease [6^▪▪^,^29^]. The general management should aim to prevent asthma exacerbations, limit airflow obstruction, and reduce the risk of asthma-related complications. Moreover, it is important to identify and appropriately manage comorbidities such as gastroesophageal reflux disease (GERD), rhinitis and sinusitis. In addition, implementing strategies to minimize exposure to allergens plays a key role in the effective management of EIB and EIA [30]. The primary long-term objective in asthma management, including in EIB and EIA, is to reduce airway inflammation and prevent bronchoconstriction, mainly using inhaled corticosteroids (ICS), administered either as monotherapy or in combination with long-acting β2-agonists (LABA). Although they are often underused compared to short-acting β2-agonists (SABA), ICS represent the cornerstone of treatment in asthmatic athletes [6^▪▪^,^30^]. Short-acting β2-agonists (SABA) provide rapid symptom relief by relaxing bronchial smooth muscle; however, their use as sole therapy in asthmatic patients is discouraged by current asthma treatment guidelines. Recent studies have shown that excessive SABA use may lead to tachyphylaxis, reducing smooth muscle responsiveness and increasing the rate of exacerbations and mortality in asthmatic patients [30,31]. Regular ICS therapy, instead, improves symptom control and enhances lung function by reducing airway responsiveness to various triggers, including exercise. Low-dose daily ICS should be initiated when a β2-agonist is required more than twice weekly or when asthma limits exercise tolerance and may be considered earlier in patients with frequent symptoms or an increased risk of exacerbations. If asthma remains uncontrolled with ICS alone, step-up therapy with the addition of a LABA is recommended [6^▪▪^,^30^]. If ICS/LABA therapy is not effective in controlling asthma symptoms and exacerbations, add-on therapies may be considered. Leukotriene receptor antagonists (LTRAs) may be administered daily as prophylaxis or 1–2 h before exercise and have been shown to prevent EIB for up to 12–24 h [19]. Another add-on option is long-acting muscarinic antagonists (LAMA). Considering the increased parasympathetic tone observed in endurance athletes, this class of add-on therapy may provide additional benefits in EIB management [8]. Furthermore, scientific evidence shows that combining pharmacologic treatment with nonpharmacologic strategies could improve control of EIB. A warm-up routine before exercise with high-intensity interval protocols can induce a refractory period lasting up to two hours, reducing bronchoconstriction in approximately 50% of athletes. Improving cardiovascular and breathing exercises may further reduce EIB symptoms. It is also crucial to control the environment: cold and dry air promotes bronchoconstriction, and strategies such as heat-exchange masks, indoor training during cold weather, and nasal breathing may reduce symptoms. Also, urban athletes should minimize their exposure to traffic pollution and adjust their training, considering air quality and pollen levels. Low-salt diets and supplementation with omega-3 fatty acids show preliminary and limited benefit. Caffeine seems to have a mild bronchodilator effect, but evidence remains limited [19], and further research is needed to determine its overall impact on athletic performance and respiratory health in urban environments.

### Special considerations

Athletes are regulated by the World Anti-Doping Agency (WADA), maintaining the integrity of sport and safeguarding health through its yearly Prohibited List. This list details substances and methods that are forbidden both during and outside of competitions. In this list, β_2_-agonists and glucocorticoids are subject to restrictions. The goal is to prevent their misuse as a means of enhancing performance. Regarding β_2_-agonists, athletes affected by EIB or asthma can utilise inhaled salbutamol, formoterol, and salmeterol without a therapeutic use exemption (TUE) if taken at a therapeutic dosage. For others inhaled β_2_-agonists like terbutaline, a TUE may be necessary. Systemic administration of any β_2_-agonist is forbidden. On the contrary, ICS are allowed without a TUE, if taken for asthma maintenance. TUEs are required for any systemic treatment during competitions, such as for worsening asthma. These rules aim to balance the health of athletes and their legal access to treatments with the goal of preventing the use of drugs to unfairly improve performance [32^▪^].

## DISCUSSION

EIB and EIA are conditions that are highly prevalent and relevant in athletes and that have implications beyond the respiratory health of these patients. The higher rate of asthma in athletes compared with the general population suggests a central role in the development of the disease of sport-specific environmental factors combined with higher ventilatory demands. A key emerging concept is the heterogeneity of airway dysfunction in athletes: while a significant proportion of them are characterized by a classical T2-high inflammatory phenotype, a substantial number of athletes present EIB in the absence of T2 biomarkers. In the latter, the pathogenesis seems to be associated with airway epithelial barrier damage caused by repetitive hyperventilation, osmotic stress, and environmental exposures (e.g., cold dry air, chlorine derivatives, pollutants). Additionally, autonomic dysregulation, particularly enhanced parasympathetic tone, may contribute to bronchial hyperresponsiveness in selected endurance athletes, suggesting that neural mechanisms deserve greater attention in future research. From a diagnostic perspective, a comprehensive evaluation of lung function, associated with anamnesis, is mandatory. Indirect bronchial provocation tests such as exercise challenges and EVH more closely reproduce the real-life mechanism of EIB compared with indirect assessments. Biomarkers like FeNO and blood eosinophils have an emerging role in EIB diagnosis, but the low sensitivity of these biomarkers demonstrated that they could not replace the provocation tests. Nevertheless, standardization of testing protocols and diagnostic thresholds is essential to avoid both overdiagnosis and underdiagnosis in this population. Differential diagnosis is also particularly relevant in master athletes, in whom cardiac conditions, chronic pulmonary diseases, anaemia, and deconditioning may mimic asthma symptoms. Upper airway disorders such as vocal cord dysfunction or structural obstruction should also be considered, especially when flow–volume loop abnormalities are present. Therapeutic management in athletes should follow the established guidelines for asthma treatment, with ICS, alone or associated with LABA, as the cornerstone of the therapy, while the overreliance on SABA should be discouraged. Early initiation of ICS and appropriate step-up strategies, including ICS/LABA combinations, LTRA and LAMA, should be guided by disease severity. The potential cardiovascular and metabolic side effects of certain drugs should be considered in the management of asthma in athletes. In fact, it is crucial to treat the disease effectively while minimising potential side effects that could impair performance. SABA and LABA are associated with adverse effects such as tachycardia, supraventricular arrhythmias, tremor, and hypokalaemia. Although systemic absorption of ICS is lower compared with oral corticosteroids (OCS), prolonged use of high doses may lead to metabolic adverse effects such as dyslipidaemia, insulin resistance, and hypertension, which are risk factors for cardiovascular disease [29,30]. Also, in athletes, in particular in elite ones, WADA regulations should be considered with attention when prescribing EIB treatment. These considerations underline the need for a personalised treatment approach for each athlete rather than a one-size-fits-all strategy. Non-pharmacological interventions also play an important complementary role. High-intensity warm-up protocols, environmental control strategies and breathing techniques may reduce symptom burden.

## CONCLUSION

In conclusion, EIA and EIB should not be considered just exercise-induced conditions, but instead, they should be viewed as multifaceted airway disorders mediated by environmental, immunological, epithelial, and autonomic factors. A more comprehensive understanding of the mechanisms driving the pathophysiology of EIB may improve phenotyping accuracy and facilitate the development of personalized diagnostic and therapeutic approaches for athletes with airway diseases.

## Acknowledgements

*None.*

### Financial support and sponsorship

*None.*

### Conflicts of interest

*There are no conflicts of interest.*

## REFERENCES AND RECOMMENDED READING

Papers of particular interest, published within the annual period of review, have been highlighted as:

▪ of special interest
▪▪ of outstanding interest
